# Virtual Reality Cognitive Remediation in Older Adults with Bipolar Disorder: The Effects on Cognitive Performance and Depression in a Feasibility Randomized Controlled Trial

**DOI:** 10.3390/healthcare12171753

**Published:** 2024-09-03

**Authors:** Diego Primavera, Cesar Aviles Gonzalez, Alessandra Perra, Goce Kalcev, Elisa Cantone, Giulia Cossu, Anita Holzinger, Mauro Giovanni Carta, Federica Sancassiani

**Affiliations:** 1 Department of Medical Sciences and Public Health, University of Cagliari, 09042 Cagliari, Italy; 2 Department of Nursing, Universidad Popular del Cesar, Valledupar 200001, Colombia; 3 The National Alliance for Neuromuscular Diseases and Neuroscience GANGLION Skopje, 1000 Skopje, North Macedonia; 4 Department at Medical, University of Wien, Spitalgasse 23, 1090 Wien, Austria

**Keywords:** cognitive remediation, virtual reality, advanced technology laboratory, elderly

## Abstract

Introduction: Dementia, depression, and cardiovascular disease are major public health concerns for older adults, requiring early intervention. This study investigates whether a virtual reality cognitive remediation program (VR-CR) can improve cognitive function and depressive symptoms in older adults, and determines the necessary sample size for future studies. Integrated VR and CR interventions have shown promising outcomes in older adults with neurodegenerative and mental health disorders. Methods: This secondary analysis of a randomized controlled trial involves adults aged 58–75 years with bipolar disorder, excluding those with acute episodes, epilepsy, or severe eye diseases. The experimental group received standard treatment plus VR-CR, while the control group received only standard treatment. Results: No baseline differences were found between the experimental and control groups. No significant improvement was observed in the overall cognitive function test (*p* = 0.897) or in depressive symptoms (*p* = 0.322). A phase III efficacy study requires a sample size of 28 participants (alpha = 0.05, beta = 0.20). Conclusions: VR-CR can potentially treat depressive symptoms in adults and older adults, but the results support conducting phase III studies to further investigate these outcomes. However, the improvement in cognitive performance in the elderly is less pronounced than in younger individuals.

## 1. Introduction

Dementia, mood disorders (depression), and cardiovascular disease are major public health issues and significantly impact older adults. Approximately 55 million people worldwide are living with dementia [1]. Estimates indicate that this number will double every 20 years. The increase is greater in developing countries, where life expectancy is rising, such as China, India, and countries in South Asia and the Western Pacific [2]. The increasing elderly population and related societal issues present significant challenges for communities. The community environments in which people are embedded act as determinants for health and disease risk [3]. According to the social determinant theoretical framework for health [4,5,6], recent findings underline the necessity for policy interventions in public health, particularly targeting high-risk groups as a strategy for reducing the incidence of dementia [3]. Among high-risk groups, it is important to note that cardiovascular diseases are linked to dementia, and alterations in cerebral perfusion can exacerbate the neurodegenerative processes associated with Alzheimer’s disease [7]. Additionally, the risk of developing dementia is higher for individuals born in states with high stroke mortality [8]. Other risk factors and subgroups of the population also need attention. Mood disorders are closely linked to both dementia and cardiovascular diseases. The concept of vascular depression [9,10], a common condition in older adults where cardiovascular and symptom disorders act as mutual risk factors and aggravate each other, explains part of this close link [11]. Although the relationship between Alzheimer’s disease and mood disorders remains unclear, it is well established that major depressive disorders and subthreshold depressive disorders can precede the onset of Alzheimer’s disease [12]. Bipolar disorder is also associated with an increased risk of cognitive impairment dementia [13,14,15] and cardiovascular diseases [16,17]. The life expectancy gap between people with bipolar disorder and the general population is largely due to excess deaths from cardiovascular disease and cognitive decline [18]. However, it is debated whether the relationship between bipolar disorder, cardiovascular disease, and dementia is identical to that observed with depressive disorders. According to the current classification of psychiatric disorders by the American Psychiatric Association (DSM-5), depressive disorders and bipolar disorders are distinctly separated based on different pathogenetic and course hypotheses [19]. This approach is less accepted in Europe, where the concept of an all-encompassing bipolar spectrum [20,21,22], which includes all mood disorders in a pyramid of non-clinical conditions characterized by hyperactivity, hyper-energy, and novelty-seeking, is still widespread among psychiatrists [23,24,25]. A non-specific syndrome characterized by alterations in social rhythms could be the intermediate stage between pathology and well-being [24,26,27].

Beyond the acceptance of the bipolar spectrum concept, it is essential to address the risks associated with cardiovascular, depressive, and bipolar disorders, as well as the life course factors influenced by location, such as health behaviors, quality of education, and experiences of discrimination, in relation to cognitive impairment among elderly people [3,28]. Given the strong associations with multifactorial risk factors, ranging from cardiovascular diseases to bipolar disorder, it is important to promote interventions that adopt a holistic approach. These interventions should aim to improve not only cognitive functions in the elderly, but also general clinical outcomes, including aspects related to well-being that influence both physical and mental health, such as depressive symptoms. The prevention and early treatment of these conditions are crucial challenges for global public health. Cognitive remediation interventions, in particular, have shown positive results not only in improving cognitive functions but also in enhancing aspects related to well-being [29].

Currently, reviews have highlighted that the use of technological innovation tools for cognitive remediation programs, such as immersive virtual reality, can be accessible and have good rehabilitative potential for elderly people [29,30]. Recently, a randomized controlled feasibility study that employed a virtual reality cognitive remediation program aimed at improving cognitive impairment in bipolar disorder showed promising results in both primary and secondary outcomes [31,32]. The study demonstrated good feasibility in terms of acceptability, tolerability, and the presence of side effects associated with immersive virtual reality (primary outcomes). Additionally, it showed improvements (secondary outcomes) in cognitive function and depressive symptoms in the general sample with different age groups. While the RCT specifically targeted individuals with bipolar disorder, the findings indicate that fully immersive virtual reality could potentially benefit broader populations in preventing cognitive decline and improving clinical outcomes such as depressive symptoms. Therefore, given the strong relationships between depression, dementia, and cardiovascular diseases in elderly populations, we aim to investigate the hypothesis that these results may also be applicable to the subgroup of older adults.

### Aims

For all of these reasons, the primary aim of this work, which is a secondary analysis of a previous study [32], is to verify whether the cognitive remediation program using virtual reality achieves similar results in a sub-sample of older adults to those observed in the entire sample, specifically in terms of cognitive impairment and depression, which are of particular interest for prevention strategies in old age. A secondary aim of this study is to calculate, based on the results, the sample size (or specific sub-sample of older adults) needed to conduct a sufficiently powered phase 3 study and to evaluate the feasibility of such a specific phase 3 study.

## 2. Materials and Methods

### 2.1. Design

This is a secondary analysis focusing on older adults (aged > 58 years) and based on the results of a randomized, controlled clinical feasibility study [31,32]. The protocol was conducted in accordance with the CONSORT flow diagram modified for feasibility studies [33].

### 2.2. Sample

Participants were recruited from the Consultation and Psychosomatic Psychiatry Unit at the “San Giovanni di Dio” Hospital in Cagliari. Inclusion criteria were as follows: aged between 58 and 75 years, with a diagnosis of bipolar disorder according to the DSM-IV [34], with no exclusion based on sex. Participants or their legal representatives had to provide signed informed consent. Exclusion criteria included the presence of an acute episode of depression or mania, a diagnosis of epilepsy, or severe eye diseases, due to the potential for excessive stimulation from virtual reality. The recruitment phase involved screening patients at the center for eligibility. Once deemed eligible, patients were recruited and randomized. Randomization followed a 1:1 allocation ratio and participants were randomized into two groups using a computer randomization method. The person responsible for the randomization was blinded to the names of the participants.

### 2.3. Control and Experimental Intervention

The experimental group received treatment as usual plus the virtual reality remediation intervention (two weekly sessions for three months). The control group received only treatment as usual, which consisted of regular psychiatric visits and pharmacotherapy. The “CEREBRUM” software version 3.0.1 used in the cognitive remediation program has been described in detail in previous articles [31,32]. In summary, CEREBRUM offers virtual scenarios simulating everyday life, consisting of 52 exercises that address various cognitive tasks and skills. The exercises increase in difficulty, but the clinician adapts the level of difficulty to the functional needs and specific abilities of the participants. This approach ensures a stimulating learning environment where exercises are neither too easy nor too difficult.

A multidisciplinary team, including psychiatric rehabilitation technicians, psychiatrists, and psychologists, carried out the intervention. To ensure the generalizability of the intervention, the sessions were well structured and defined in previous studies, following a recovery-oriented and person-centered model [35,36,37].

### 2.4. Data Collection and Instruments

In the present study, cognitive performance levels and depressive symptoms were examined. These levels were measured before treatment (T0) and after treatment (T1). Standardized tools validated in Italian and commonly used in mental health research were utilized for all variables.

Cognitive performance was evaluated using the following tools and areas: Visuospatial functions were measured by using the Rey–Osterrieth Complex Figure Test [38]. Memory functions were measured through immediate and delayed recall of Rey’s Words Test [39,40], the Story Recall Test [41], and the Backward Digit Span [42,43]. Attention was measured by using the Forward Digit Span [42,43], the Trail Making Test Part A [40,44], and the Matrix Test [40]. Language was measured by using the Phonological and Semantic Verbal Fluency Test [41,45]. Executive functions were measured by using the Trail Making Test Part B [46,47], the Stroop Test [38], the Digit Symbol Substitution Test [46,47], the Frontal Assessment Battery (FAB) [48], and the Cognitive Estimates Test (CET) [49,50].

Depressive symptoms were evaluated using the Patient Health Questionnaire-9 (PHQ-9) [51], a self-administered assessment scale translated into the Italian version [52]. This tool identifies depressive episodes based on the main symptoms defined in the Diagnostic and Statistical Manual of Mental Disorders (DSM-5). According to international cut-off scores, individuals with a PHQ-9 score >15 are considered to have a severe depressive episode [51].

### 2.5. Statistical Analysis

This feasibility study was conducted using a sample size that did not allow for post-analysis with multivariate techniques to test hypotheses in the sub-sample of older adults. Therefore, the present analysis was conducted using non-parametric statistical tests. The effect of the intervention was determined by measuring and comparing the conditions in both the experimental and control groups. Specifically, we assessed the transition from a deficient condition before the intervention to a below cut-off condition after the intervention—conditions that remained unchanged—and the transition from an above cut-off condition to a deficient or below cut-off condition. This measurement was conducted using the Kruskal–Wallis test, considering a total of 15 tests on cognitive functions (Rey Immediate Score cut-off = 6.44; Frontal Assessment Battery cut-off = 15.7; Digit Symbol Substitution cut-off = 34.2; all other tests’ cut-off = 0), and the PHQ-9 for depression (cut-off = 15). The effect on individual cognitive tests was assessed by evaluating the improvements and increases in cut-off scores and comparing the two groups using the Fisher exact test. The sample size (or specific sub-sample of older adults) needed to conduct a sufficiently powered phase 3 study was calculated based on the results, with alpha = 0.05, beta = 0.20, and power = 0.80.

## 3. Results

Table 1 illustrates the sample characteristics in the two study arms. The results of the performance tests indicate a low average level, with the majority of tests showing a standard deviation very close to the cut-off, indicating dysfunction. Notably, for all tests with a numerical cut-off different from 0, the standard deviation was lower than the cut-off (Rey Immediate Score, Digit Symbol Substitution); the average performance was even below the cut-off (FAB). The scores achieved on the PHQ-9 (depression) also indicate a sample with dysfunctional and suffering characteristics. The average PHQ-9 score was very close to the cut-off for severe depression. No baseline differences emerged between the experimental and control samples in any of the measurements used.

Table 2 illustrates how, using the measures considered, there were individuals whose responses to the PHQ-9 or single results in one of the performance tests (out of the 15 tests) transitioned from below the cut-off to above, or vice versa, following the virtual reality remediation intervention. Considering the three levels of ranking (improved, unchanged, worsened), the difference between the experimental group and the control group was assessed using the Kruskal–Wallis test. This analysis highlighted a fair improvement in the PHQ-9, although the difference did not reach statistical significance. However, there was an improvement of over 25% compared to the control group. In contrast, the advantage achieved in the performance tests not only did not result in a statistically significant difference, but it was just over 10%. Out of the total of dysfunctional tests in the experimental group at baseline, an improvement of 65% was achieved, compared to only 2 trials becoming dysfunctional (0.89% of previously functional trials). In the control group, 10 trials became functional out of 18 dysfunctional ones (55%), and 2 observations became dysfunctional, representing 2.22% of trials previously above the cut-off. As a result, the calculation of the necessary sample size for conducting an efficacy study in phase 3 (with alpha = 0.05 and beta = 0.20) indicates the need for a sample of 68 participants for the evaluation of the effectiveness of the virtual reality cognitive remediation path in the PHQ-9, and 628 subjects for cognitive performance. The change in cognitive tests before and after the intervention in the two groups is reported in the Table S1 (Supplementary Materials). No statistically significant difference in improvement was observed when comparing the experimental group with the control group in any of the tests considered. concerning visuospatial (Rey–Osterieth Complex Figure Test), attention (Forward Digit Span TMT part A, and Matrix Test), memory (immediate and delayed recall of Rey’s Words Test, the Story Recall Test, and the Backward Digit Span), language (Phonological and Semantic Verbal Fluency Test), and executive functions (TMT part B, Stroop Test, DSST, FAB, and CET).

## 4. Discussion

This study shows that in a small sample of elderly adults with bipolar disorder, characterized by cognitive performance at the limits of normality (but with numerous performance tests below normal), and a strong presence of depressive symptoms, an intervention of cognitive remediation with virtual reality could reduces the number of people with severe depression in the experimental group, with a difference compared to the control group that does not reach statistical significance but is large enough to strongly encourage conducting a phase III study.

The result is not as positive in relation to the cognitive performance tests, which improve only slightly in the experimental group, without achieving a statistically significant difference compared to the control group when considering either the total performances or the individual tests. This result does not confirm the findings from the analysis of the entire sample, which included younger participants and involved 64 observations, compared to only 21 in the current elderly sample [32]. This discrepancy necessitates reflection on the specific negative result in the elderly group. It could be attributed to either greater generational challenges with unfamiliar digital tools and internet usage or to potentially different stages of cognitive impairment development in bipolar disorder, where the intervention may be more effective. However, the promising results on the PHQ-9, although not statistically significant in the small sample of elderly adults, align with the results achieved in the entire study sample that included younger participants, indicating greater statistical power.

If these results are confirmed in larger samples, two distinct causes for the development of cognitive impairment in bipolar disorder could be hypothesized. The first cause could be an excess of cardiovascular disease, aligning with a neurodegenerative hypothesis [7,9,10]. The second cause might be due, in part or entirely, to concomitant metabolic disorders (largely due to medication) [53,54], a sedentary lifestyle [55], and frequent substance abuse [56,57], all of which are associated with bipolar disorder. This question remains to be answered. A study analyzing a large cohort of 700,000 individuals with various psychiatric disorders found that bipolar disorder is associated with an elevated risk of developing cerebrovascular and neurodegenerative disorders compared to other major psychiatric disorders, including major depressive disorder [11,58]. The authors suggested that this might support the “neuroprogressive view of bipolar disorder”. However, although the cohort had a low average age (around 40 years), the study did not specify whether those with cerebrovascular disease were older on average than those with bipolar disorder without cerebrovascular disease [58]. Other studies have indicated that the risk of neurovascular events in bipolar disorder accumulates over time, increasing in adults and older adults [59]. This is consistent with the excess mortality and the gap of about 10–15 years in life expectancy in people with bipolar disorder compared to the general population, largely due to cardiovascular disorders [17,18] or chronic diseases involving cardiovascular compromise [60,61].

If this hypothesis is confirmed, it would have significant clinical relevance. Cognitive impairment episodes linked to cardiovascular risk in people with bipolar disorder are more likely to be difficult to treat with cognitive remediation alone. Therefore, rehabilitative interventions should include integrated approaches addressing other dimensions of psychological well-being and functioning [62,63].

However, another area of research on cognitive impairment, mainly involving individuals with juvenile or young adult bipolar disorder, has focused on social competence tasks and so-called social intelligence [64,65,66,67,68,69]. People with bipolar disorder, even during the euthymic phase and at a young age, exhibit difficulties in social cognition [64,65]. Social intelligence involves both cognitive and affective skills [66,67,68]. In bipolar disorder, difficulty in mentalizing, i.e., understanding the emotions and intentions of oneself and others, has been observed [67,68]. When faced with emotional problems requiring complex responses, individuals with bipolar disorder show low activation of the “mirror neuron system” (premotor cortex, right inferior frontal cortex, insula) [65]. This results in a “fast” and “inefficient” response method that relies on cognitive processes without the necessary integration of emotional and cognitive responses. The promising results achieved by virtual reality, particularly in younger individuals, but not detectable in the elderly, may specifically relate to these social cognitive abilities which are “intrinsic” to the disorder and are not a consequence of cardiovascular risk and subsequent cardiovascular dementia.

## 5. Conclusions

Our study found that even in adults and older adults, cognitive remediation by means of virtual reality could play a role in the therapy of depressive symptoms. The results of our study encourage phase III studies regarding these outcomes. However, in the elderly and older adults, the result for cognitive performance is not as brilliant as in young people. Further studies will have to clarify the divergence of the results at different ages and verify the hypothesis of a double type of cognitive deficit in bipolar disorder, with the first one being characteristic of social skills, intrinsic to the disorder and independent of age, and the second one being associated with vascular disorders.

## Figures and Tables

**Table 1:** The characteristics of the sample and the scores of the outcome measures before the trial.

	Experimental Group *N* = 15	Control Group *N* = 6	Statistics
Sex (Female)	11 (78.6%)	4 (66.7%)	Fisher exact test *p* = 0.802
Age	61.53 ± 3.57	61.33 ± 2.49	H = 0, *p* = 1 (KW)
Rey Immediate (cut-off = 6.44)	10.02 ± 7.06	8.01 ± 6.42	H = 0.387, *p* = 0.533 (KW)
Rey’s Words Immediate (cut-off = 0)	2.06 ± 1.69	2.16 ± 1.86	H = 0.054, *p* = 0.805 (KW)
Ray’s Words Delayed (cut-off = 0)	2.10 ±1.45	2.66 ± 1.24	H = 0.097, *p* = 0.755 (KW)
Stroop Test Time (cut-off = 0)	2.40 ± 1.66	2.33 ± 1.69	H = 0.024, *p* = 0.876 (KW)
FAB (cut-off = 15.7)	14.06 ± 3.35	13.16 ± 2.79	H = 0.490, *p* = 0.483 (KW)
Digit Span Direct (cut-off = 0)	2.26 ± 1.43	2.58 ± 1.60	H = 0.183, *p* = 0.668 (KW)
Digit Span Backward (cut-off = 0)	1.80 ± 1.37	1.66 ± 1.49	H = 0.054, *p* = 0.815 (KW)
Verbal Phonological Test (cut-off = 0)	2.10 ± 1.45	2.00 ± 1.73	H = 0.097, *p* = 0.755 (KW)
Verbal Semantic Test (cut-off = 0)	2.33 ± 1.44	2.16 ± 1.67	H = 0.038, *p* = 0.845 (KW)
Matrix (cut-off = 0)	1.73 ± 1.43	2.16 ± 1.67	H = 0.340, *p* = 0.559 (KW)
DSST (cut-off = 34.2)	36.23 ± 14.30	36.06 ±14.42	H = 0.038, *p* = 0.845 (KW)
CET (cut-off = 0)	1.93 ± 1.34	2.33 ± 0.74	H = 0.341, *p* = 0.559 (KW)
Test of Tale (cut-off = 0)	1.60 ± 1.26	1.66 ± 1.24	H = 0.297, *p* = 0.586 (KW)
TMT-A (cut-off = 0)	2.66 ± 1.39	2.66 ± 1.49	H = 0.006, *p* = 0.937 (KW)
TMT-B (cut-off = 0)	2.73 ± 1.57	2.83 ±1.67	H = 0.218, *p* = 0.640 (KW)
PHQ-9 (cut-off = 15)	14.93 ± 5.89	11.16 ± 4.94	H = 1.274, *p* = 0.258 (KW)

**Table 2:** Efficacy considering all 15 cognitive tests and depressive disorders according to PHQ9. Sample size for phase III study with alpha of 0.05, beta of 0.2, and power of 0.8.

		T0–T1 EG (*N* = 15)	T0–T1 CG (*N* = 6)	Kruskall–Wallis	Sample Size
All 15 cognitive tests	Improved (people/observations with scores below the cut-off who exceed the cut-off after the trial)	23	10 (55.5%)	H = 0.017 *p* = 0.897	628 (314 by arm)
	Unchanged (people whose score level is below threshold and above threshold do not change during the trial)	200	78		
	Worsened (people with scores above the cut-off whose scores decrease below the cut-off after the trial)	2 (0.89%)	2 (2.22)		
PHQ-9	Improved	4 (26.6%)	0 (0 s)	H = 0.976 *p* = 0.322	68 (34 by arm)
	Unchanged	11	6		

## Data Availability

All data generated or analyzed during this study are included in this published article.

## Supplementary Materials

The following supporting information can be downloaded at https://www.mdpi.com/article/10.3390/healthcare12171753/s1, Table S1: Results of specific test on cognitive performance.
