# Supplementary material for: Virtual Reality Cognitive Remediation in Older Adults with Bipolar Disorder: The Effects on Cognitive Performance and Depression in a Feasibility Randomized Controlled Trial
Source: Healthcare (Basel). 2024 Sep 3;12(17):1753. doi: 10.3390/healthcare12171753 (PMC11394966; doi:10.3390/healthcare12171753)
Supplement: Supplementary file 1 [file healthcare-12-01753-s001.zip › healthcare-3179026-supplementary.pdf]

Table S1. Results on specific test on cognitive performance

	T0	T1	Improved (%; Fisher Exact test)
Fig. Rey Immediate EX (N=15)	6	4 (25%)	2 (33.3%)
Fig. Rey Immediate CC (N=6)	3	1 (16.7%)	2 (33.3%)
			p=0.292
Rey's Words Immediate (N=15) EX	5	2	3 (60%)
Rey's Words Immediate (N=6) CC	2	2	0 (0%)
			p=0.531
Ray's Words Delayed (N=15) EX	2	2	0 (0%)
Ray's Words Delayed (N=6) CC	0	1	-1(-16.7%)
			p=0.273
Stroop Test Time (N=15) EX	4	2	2 (50%)
Stroop Test Time (N=6) CC	2	2	0 (0)
			p=0.519
Frontal Assesment Battery (N=15) EX	9	8	1 (11.1%)
Frontal Assesment Battery (N=6) CC	5	3	2 (40%)
			p=0.169
Digit Span Direct EX (N=15)	2	1	1 (50%)
Digit Span Direct CC(N=6)	1	0	1 (100%)
			p=0.481
Digit Span Backward (N=15) EX	3	5	-2 (16.7%)
Digit Span Backward (N=6) CC	2	2	0 (0)
			p=0.571
Verbal Phonological Test (N=15) EX	3	1	2 (33.3%)
Verbal Phonological Test (N=6) CC	2	1	1 (50%)
			p=0.636
Verbal Semantic Test (N=15) EX	2	0	2 (100%)
Verbal Semantic Test (N=6) CC	2	1	1 (50%)
			p=0.636
Matrix (N=15) EX	5	4	1 (20%)
Matrix (N=6) CC	2	1	1 (50%)
			p=0.481
Substit. Digit Symbol (N=15) EX	9	6	3 (30%)
Substit. Digit Symbol (N=6) CC	2	3	-1 (-20%)
			p=0.364
Cog. Estimation Test (N=15) EX	3	0	3 (100%)
Cog. Estimation Test (N=6) CC	0	0	0 (0)
			p=0.531
Test of Tale (N=15) EX	2	1	1 (50%)
Test of Tale (N=6) CC	1	0	1 (100%)
			p=0.481
Trial Making Test PartA (N=15) EX	2	2	0 (0)
Trial Making Test PartA (N=6) CC	1	0	1 (100%)
			p=0.273
Trial Making Test Partb (N=14) EX	3	1	2 (66.7%)
Trial Making Test Partb (N=6) CC	1	1	0 (0)
Total	60	18	p=0.519
